# Two-dimensional echocardiographic and strain values of the proximal thoracic aorta in a normal sub-Saharan African population

**DOI:** 10.1186/s44156-023-00016-x

**Published:** 2023-02-15

**Authors:** Ruchika Meel, Kelly Blair

**Affiliations:** 1 Division of Cardiology, Department of Internal Medicine, Chris Hani Baragwanath Academic Hospital, Faculty of Health Sciences, University of the Witwatersrand, 7 York Rd, Parktown, Johannesburg, 2193 South Africa; 2 Chris Hani Baragwanath Academic Hospital, Faculty of Health Sciences, University of the Witwatersrand, Johannesburg, South Africa

**Keywords:** Africa, Normal African population, Echocardiography, Aorta dimensions and strain

## Abstract

**Background:**

There is limited data regarding reference ranges for aortic dimensions in African populations. This study aims to establish normal reference ranges for echocardiographic dimensions and circumferential strain (CS) of the proximal thoracic aorta in a healthy sub-Saharan African population.

**Methods:**

This was a secondary analysis of data from a prospective cross-sectional study of 88 participants conducted at Chris Hani Baragwanath Hospital (2017–2019). Aortic measurements were obtained as per the 2015 American Society of Echocardiography guidelines using a Philips iE33 system. Circumferential Strain was measured using Philips QLAB version 11.0 software offline semi-automated analysis of speckle-based strain 2-D speckle-tracking software (Amsterdam, The Netherlands).

**Results:**

Mean age was 37.22 ± 10.79 years (41% male). The mean diameter at the aortic annulus, sinuses, sino-tubular junction (STJ) and ascending aorta (AAO) were 19.11 ± 2.38 mm, 27.40 ± 6.11 mm, 25.32 ± 3.52 mm and 25.36 ± 3.38 mm, respectively. Males had larger absolute and indexed aortic diameters at all levels when compared to females. The mean aorta CS was 11.97 ± 5.05%. There was no significant difference in CS based on gender (12.19 ± 5.04% vs 11.51 ± 5.02%, P = 0.267). On multivariate linear regression analysis, male sex was the most significant predictor of increased diameter at the level of the aortic annulus (r = 0.17, P = 0.014), body surface area was the most significant predictor at the sinuses (r = 0.17, P = 0.014) and AAO (r = 0.30, P < 0.001), while age was the most significant predictor at the STJ (r = 0.27, P = 0.004). There was a negative correlation between age and aortic CS (r = − 0.12, P < 0.001). The most important predictor of aorta CS was age, on multivariate analysis (r = − 0.19, P = 0.024).

**Conclusions:**

This study provides normal reference ranges for dimensions of the proximal aorta and circumferential strain (CS) in a sub-Saharan African population according to age, sex, and body habitus. It serves as a platform for future larger studies and allows for risk stratification of cardiovascular disease in an African population.

## Introduction

Aortic dilatation is a decisive predictor of morbidity and mortality in aortic and cardiovascular disease. The greater the dilatation of the aorta, the greater the risk for aortic rupture and dissection [1]. Transthoracic echocardiography is universally used for imaging of the proximal thoracic aorta and consequently is used as a screening tool for aortic dilatation and aneurysm [2]. Accurate and timely echocardiographic screening is required to prevent progression from aneurysm to aortic rupture or dissection [2]. For screening to be effective it is necessary to understand the normal values for aortic diameters in healthy patients. Reference values for echocardiographic aortic measurements were published by the American Society of Echocardiography (ASE) and the European Association of Echocardiography (EAE) in 2015 [3]. While these guidelines are an important advance in quantitative echocardiography, these reference values were produced based on measurements performed on predominantly Caucasian populations.

Proximal thoracic aortic dimensions are known to be age, sex, weight, and height dependent [1]. Further to this, there is evidence from studies having been done in the United States of America [4, 5], Europe (the largest being the EACVI NORRE study) [1, 6], China (The Echocardiographic Measurements in Normal Chinese Adults—EMINCA) [7], Japan (The Japanese Normal Values for Echocardiographic Measurement Project—JAMP) [8] and Korea (The Normal Echocardiographic Measurements in a Korean population- NORMAL study) [9] suggesting that there may be clinically significant differences in the sizes of the aortic diameter in people of different ethnicities [2]. Currently, there are no recorded normative echocardiographic data that either includes patients of African descent or have been performed in Africa, which can be used as a reference for patients of African descent with disease in the ascending aorta.

In addition to screening for aortic dilatation, advanced echocardiography can be used to determine aortic circumferential strain (CS). Strain is defined as the ratio of change in length in relation to the original length. CS Strain is a measure of arterial stiffness and is calculated as the change in length along the circumferential axis of the aorta during the cardiac cycle. A decrease in CS strain occurs prior to clinically apparent cardiovascular disease and so CS is increasingly being considered as an additional tool for detecting, predicting, and ultimately preventing cardiovascular disease [10].

This study aims to provide the normal reference ranges for dimensions of the proximal aorta and circumferential aortic strain for a healthy Southern African adult population and to serve as a platform for future studies and risk stratification of aortic and cardiovascular disease.

## Methods

### Study population

This was a secondary analysis of a prospective cross-sectional study of 88 participants that were recruited as healthy controls for a prior study conducted at Chris Hani Baragwanath Academic Hospital (2017–2019). Thirty- five participants were excluded as they failed to meet the study’s inclusion criteria. Study participants were recruited as volunteers who presented themselves to the echocardiography laboratory following an advertisement about the study. The inclusion criteria were participants (i) over the age of 18 years old, (ii) with no known history or symptoms of cardiovascular or lung disease, (ii) with normal blood pressure (≤ 140/90 mmHg), (iii) with an absence of diabetes or dyslipidaemia, (iv) with no ongoing or previous medical treatment, (v) in sinus rhythm (heart rate between 50 and 85 bpm). The exclusion criteria were participants (i) with abnormal electrocardiograms and (ii) suboptimal image quality of the aorta.

The study was conducted in accordance with the Declaration of Helsinki (as revised in 2013) available at: https://www.wma.net/wp-content/uploads/2016/11/DoH-Oct2013-JAMA.pdf. Ethics approval for the study was obtained from the University of the Witwatersrand ethics committee (M200977).

### Echocardiographic examination

Aortic measurements were obtained as per the 2015 American Society of Echocardiography guidelines using a Philips iE33 system. Circumferential Strain (CS) of the ascending aorta (AAO) was measured using Philips QLAB version 11.0 software allowed offline semi-automated analysis of speckle-based strain two-dimensional speckle-tracking software (Amsterdam, The Netherlands).

Transthoracic echocardiographic examinations were performed on all patients in the left lateral position. An S5-1 transducer on a Philips iE33 system was used to obtain the aortic measurements from parasternal long axis views, where the aortic root and proximal aorta, as well as the left ventricle (LV), could be visualised and measurements at four different levels in the proximal aorta could be made namely (i) the aortic annulus (AA); (ii) sinuses of Valsalva (SV); (iii) sino-tubular junction (STJ); and (iv) the proximal ascending aorta (AAO). From the same window, with appropriate probe rotation, two-dimensional short-axis views at the level of the aortic valve plane were acquired and the image depth and the sector width were adjusted to optimize proximal aorta visualization. Zoomed-in images of both left ventricle outflow tract (LVOT) in the parasternal long-axis view and of the aortic valve in the parasternal short-axis view were obtained and recorded.

As recommended by the 2015 American Society of Echocardiography (ASE) Guidelines, the aortic annulus was measured at mid-systole from inner edge to inner edge. All other aortic root measurements (i.e., maximal diameter of the sinuses of Valsalva (SV), the sino-tubular junction (STJ), and the proximal ascending aorta (AAO) were be made at end-diastole (QRS complex onset), in a leading-edge-to-leading-edge convention [11].

To determine the circumferential strain (CS) using two-dimensional (2D) speckle-tracking (ST) echocardiography, images of the ascending aorta were first obtained in the long-axis parasternal view. These images were taken at 60–80 frames/s. We used STE software to measure the CS of the aorta. Akin to measuring CS of the ventricle in short axis view a loop was manually drawn along the inner edge of the aortic wall during systole and then an additional loop near the outer edge of the aortic wall was automatically generated by the software. The software then divided the aortic wall image into six equally sized segments and the global circumferential ascending aortic strain was calculated as the mean value of the peak CS of the six segments. The data was then transferred and analysed offline using the Xcelera workstation (Philips).

### Statistical analysis

All computations for this data were carried out using Microsoft Excel (2019). All continuous variables were summarized as a mean with a standard deviation (SD) or as a median with interquartile ranges. The upper limits of data parameters were defined as the 95th percentile. The unpaired T-test was used for normally distributed variables, or the Mann–Whitney U-test for otherwise. Pearson’s correlations were used to analyse the relationships between two quantitative, continuous variables. Further, one-way ANOVA tests were used to assess the relationship between the means of the four age groups in each of the categories measured. Finally, multivariate linear regression analysis was performed to assess the effect of various variables on aortic root diameter and aortic circumferential strain. Univariate and multivariate linear regression analyses were used to identify possible independent determinants of aortic diameter and aortic circumferential strain. The independent variables with a p-value of ≤ 0.1 on univariate analysis and variables that had clinical significance were tested in the multivariate model.

## Results

### Demographic data

The clinical characteristics of the study population are summarised in Table 1. The study population was divided into four different age groups, (i) Group 1: ≤ 29 years old, consisting of 26 participants, (ii) Group 2: 30–39 years old, consisting of 25 participants, (iii) Group 3: 40–49 years old, consisting of 22 participants and (iv) Group 4: ≥ 50-year-olds, consisting of 15 participants.

**Table 1:** Clinical characteristics of the study population

Variable	Total	Male	Female	p value	Group 1	Group 2	Group 3	Group 4	P-value
	N = 88	N = 34	N = 54		≤ 29 (N = 26)	30–39 (n = 25)	40–49 (n = 22)	≥ 50 (n = 15)	
Age (years)	37.22 ± 10.79	34.46 ± 10.20	38.85 ± 10.87	0.029	24.93 ± 2.89	34.64 ± 2.78	44.55 ± 2.86	54 ± 3.72	< 0.001
Height (cm)	160.22 ± 7.41	166.00 ± 5.12	156.4 ± 6.10	< 0.001	161.63 ± 8.27	160.12 ± 6.51	161.82 ± 6.31	155.14 ± 7.17	0.032
Weight (kg)	77.52 ± 17.02	72.33 ± 12.59	80.94 ± 18.74	0.006	69.61 ± 15.94	78.52 ± 15.97	81.5 ± 18.97	84.71 ± 12.77	0.005
BSA (m^2^)	1.85 ± 0.21	1.82 ± 0.17	1.86 ± 0.23	0.155	1.76 ± 0.20	1.86 ± 0.19	1.9 ± 0.22	1.91 ± 0.17	0.045
BMI (kg/m^2^)	30.38 ± 7.23	26.25 ± 4.42	33.11 ± 7.46	< 0.001	26.84 ± 6.99	30.71 ± 6.35	31.27 ± 7.94	35.23 ± 4.80	0.003
SBP (mmHg)	126.41 ± 11.93	126.47 ± 12.68	126.38 ± 11.55	0.486	123.65 ± 11.74	126.92 ± 14.67	127.41 ± 8.93	129.07 ± 11.17	0.522
DBP (mmHg)	79.3 ± 10.62	78.38 ± 12.73	79.89 ± 9.09	0.276	76.77 ± 11.12	79.16 ± 12.03	80.77 ± 9.10	81.93 ± 9.09	0.436
Heart Rate (bpm)	72.64 ± 12.29	66.24 ± 10.47	76.75 ± 11.67	< 0.001	70.23 ± 15.61	74.36 ± 9.73	72.45 ± 12.14	74.36 ± 9.87	0.630

*BMI* body mass index, *BSA* body surface area, *DBP* diastolic blood pressure, *SBP* systolic blood pressure

### Dimensions of the proximal aorta

**Fig. 1:**
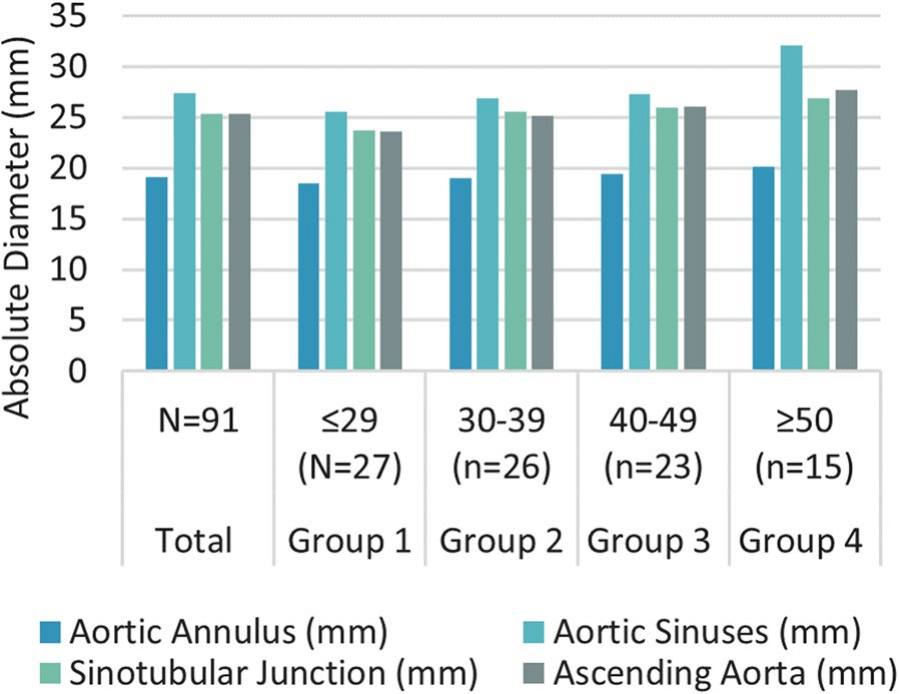
A bar graph depicting mean diameters (absolute values) of aortic annulus (AA), sinuses, sino-tubular junction (STJ), and ascending aorta (AAO) by age group

**Fig. 2:**
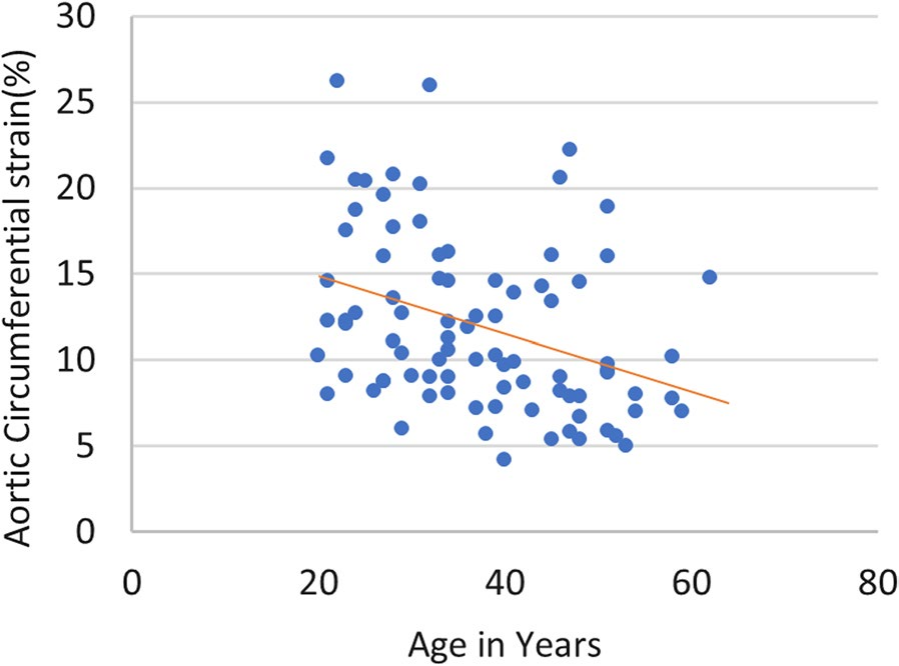
A scatterplot depicting the relationship between aortic circumferential strain and age (r = −0.17, P < 0.001)

**Fig. 3:**
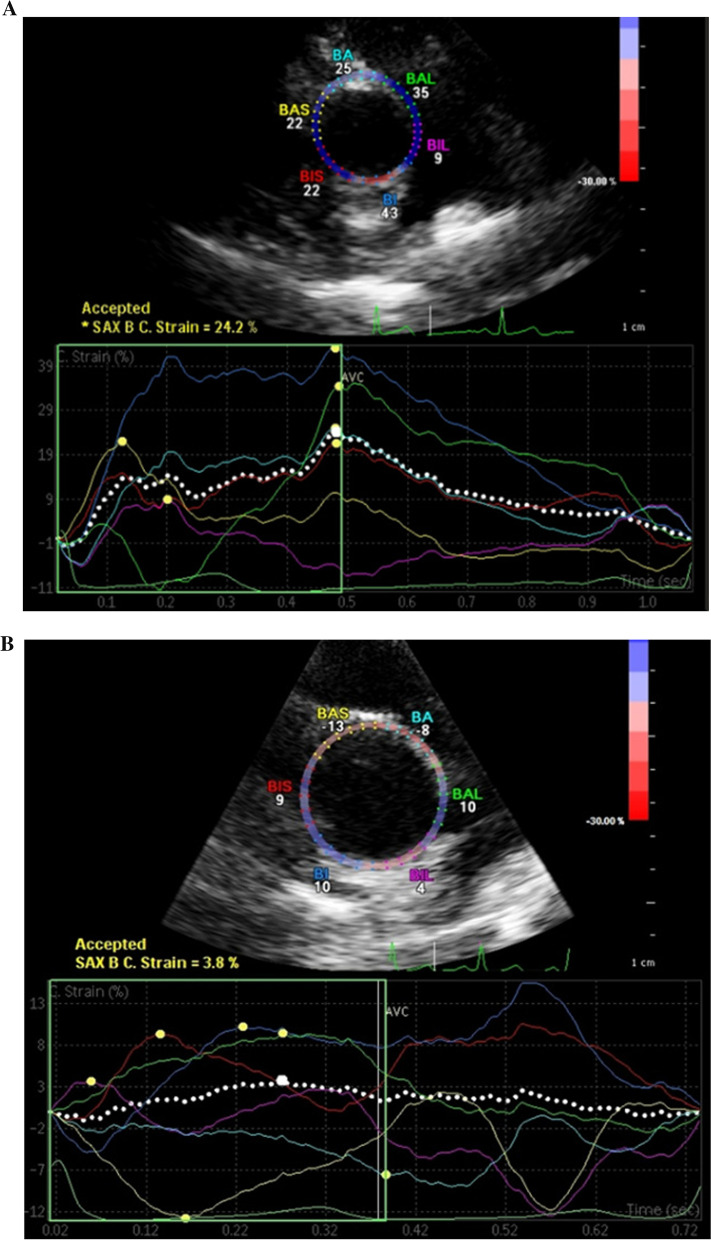
Diagram depicting a preserved aorta circumferential strain (CS) in a young patient (24.2%) (**A**) and decreased aortic CS in an older patient (3.5%) (**B**)

The baseline dimensions and echocardiographic characteristics of the proximal aorta of the study population are summarised in Table 2 and Fig. 1. For the measurements of the aortic annulus (AA), aortic sinuses, sinotubular junction (STJ) as well as the proximal ascending aorta (AAO), men had larger absolute and indexed aortic diameters than women. At the level of the aortic annulus, the difference between the male and female indexed dimensions was statistically significant at 10.87 ± 1.21 mm/m^2^ in men and 10.15 ± 1.53 mm/m^2^ in women (P < 0.001). Comparing age groups, the absolute aortic diameters at the levels of the AA, sinuses, STJ and AAO all increased with increasing age and all the indexed aortic dimensions increased with age at the significant level (P < 0.001). There was a negative correlation between age and aortic circumferential strain (r = – 0.17, P < 0.001) (Figs. 2, 3).

**Table 2:** Echocardiographic measurements of the population according to sex and age

Variable	Total	Male	Female	P value	Group 1	Group 2	Group 3	Group 4	P value
	N = 88	N = 34	N = 54		≤ 29 (N = 26)	30–39 (n = 25)	40–49 (n = 22)	≥ 50 (n = 15)	
AA (mm)	19.11 ± 2.38	19.69 ± 1.99	18.71 ± 2.54	0.028	18.5 ± 2.55	18.99 ± 2.06	19.39 ± 2.41	20.14 ± 2.28	0.188
AA/BSA (mm/m^2^)	8.99 ± 1.12	10.87 ± 1.21	10.15 ± 1.53	< 0.001	10.59 ± 1.45	10.3 ± 1.48	9.12 ± 1.13	10.83 ± 1.23	< 0.001
Sinuses (mm)	27.4 ± 6.11	28.08 ± 3.62	26.89 ± 7.34	0.044	25.53 ± 3.57	26.9 ± 2.49	27.3 ± 4.12	32.07 ± 12.24	0.01
Sinuses/BSA (mm/m^2^)	12.89 ± 2.87	15.50 ± 2.02	14.49 ± 3.16	0.053	14.63 ± 2.02	13.35 ± 1.24	12.84 ± 1.94	17.24 ± 6.58	< 0.001
STJ (mm)	25.32 ± 3.52	26.23 ± 3.38	24.67 ± 3.49	0.001	23.74 ± 4.50	25.56 ± 1.94	26 ± 2.99	26.86 ± 3.35	0.024
STJ/BSA (mm/m^2^)	11.91 ± 1.65	14.47 ± 1.84	13.36 ± 1.89	0.132	13.57 ± 2.34	12.69 ± 0.96	12.23 ± 1.41	14.43 ± 1.80	0.001
AAO (mm)	25.36 ± 3.38	25.66 ± 3.02	25.04 ± 3.58	0.268	23.63 ± 3.56	25.16 ± 2.59	26.09 ± 2.91	27.71 ± 3.34	0.001
AAO/BSA (mm/m^2^)	11.93 ± 1.59	14.15 ± 1.84	13.55 ± 1.92	0.369	13.51 ± 1.85	12.49 ± 1.29	12.27 ± 1.37	14.89 ± 1.79	< 0.001
Aorta CS (%)	11.97 ± 5.05	11.51 ± 5.02	11.38 ± 6.71	0.051	13.99 ± 5.27	12.20 ± 4.62	10.52 ± 4.85	9.62 ± 4.16	0.427

*AA* aortic annulus, *AAO* ascending aorta, *CS* circumferential strain, *STJ* sinotubular junction, *Sinuses* aortic sinuses

### Circumferential strain of the ascending aorta

The mean aortic circumferential strain (CS) for the study population was 11.97 ± 5.05%, with no significant difference in CS between men and women in the study (11.51 ± 5.02% vs 11.38 ± 6.71, P = 0.051). When comparing the different age groups, there was a decrease in aortic CS with increasing age group (P = 0.427) (Table 2; Fig. 3).

### Predictors of proximal aorta dimensions

On Univariate linear regression analysis (Table 3) for the aortic annulus and sinotubular junction age, sex, BMI and BSA were predictors of increased diameters and were included in the multivariate model. For the aortic sinuses and the ascending aorta only age, BSA and BMI were significant predictors in univariate analysis and so included in the multivariate analysis. On multivariate linear regression analysis, male sex was the most significant predictor of increased diameter at the level of the aortic annulus (r = 0.15, P = 0.037). BSA was the most significant predictor of increased diameters at the level of the sinuses (r = 0.17, P = 0.014), the STJ (r = 0.27, P = 0.015) and at the AAO (r = 0.30, P < 0.001) (Table 3).

**Table 3:** Univariate and multivariate linear regression analysis for Aortic Diameters

Univariate analysis			Multivariate analysis		
Aortic annulus			Model 1 aortic annulus: R = 0.15 P = 0.0015		
Variables	β-Coefficient ± SE	P-value	Variables	β-Coefficient ± SE	P-value
Age (years)	0.05 ± 0.02	0.024	Age (years)	0.04 ± 0.02	0.109
BSA (m^2^)	3.80 ± 1.22	0.003	BSA (m^2^)	3.15 ± 2.40	0.193
BMI (kg/m^2^)	0.064 ± 0.03	0.071	BMI (kg/m^2^)	0.01 ± 0.08	0.885
Male	0.96 ± 0.52	0.071	Male sex	1.39 ± 0.65	0.037

Aortic sinuses			Model 2 aortic sinuses: R = 0.17 P = < 0.001		
Variables	β-Coefficient ± SE	P-value	Variables	β-Coefficient ± SE	P-value
Age (years)	0.21 ± 0.06	0.001	Age (years)	0.15 ± 0.06	0.016
BSA (m^2^)	0.15 ± 3.08	0.001	BSA (m^2^)	8.09 ± 3.22	0.014
BMI (kg/m^2^)	0.149 ± 0.09	0.106			
Male sex	1.20 ± 1.37	0.383			

Sinuotubular junction			Model 3 sinotubular junction: R = 0.27 P < 0.001		
Variables	β-Coefficient ± SE	P-value	Variables	β-Coefficient ± SE	P-value
Age (years)	0.12 ± 0.03	0.001	Age (years)	0.10 ± 0.03	0.004
BSA (m^2^)	6.84 ± 1.76	< 0.001	BSA (m^2^)	8.22 ± 3.30	0.015
BMI (kg/m^2^)	0.103 ± 0.05	0.051	BMI (kg/m^2^)	− 0.09 ± 0.11	0.383
Male sex	1.55 ± 0.77	0.049	Male sex	1.76 ± 0.89	0.054

Ascending aorta			Model 4 ascending aorta: R = 0.30 P < 0.001		
Variables	β-Coefficient ± SE	P-value	Variables	β-Coefficient ± SE	P-value
Age (years)	0.14 ± 0.03	< 0.001	Age (years)	8.11 ± 3.34	0.018
BSA (m^2^)	7.61 ± 1.63	< 0.001	BSA (m^2^)	9.19 ± 2.65	< 0.001
BMI (kg/m^2^)	0.155 ± 0.04	0.001	BMI (kg/m^2^)	9.19 ± 2.65	0.085
Male sex	0.61 ± 0.76	0.418			

*BMI* body mass index, *BSA* body surface area, *DBP* diastolic blood pressure, *SBP* systolic blood pressure

### Predictors of proximal aorta circumferential strain

On Univariate linear regression analysis (Table 4) age, as well as increased diameter of the aortic annulus, sinotubular junction and ascending aorta were independent predictors of decreasing proximal aortic strain. On multivariate analysis, age was the most important independent predictor of aortic circumferential strain (r = − 0.19, P = 0.024) (Table 4).

**Table 4:** Univariate and multivariate analysis of aortic circumferential strain

Univariate analysis			Multivariate analysis R = 0.19 P < 0.001		
Variables	β-Coefficient ± SE	P-value	Variables	β-Coefficient ± SE	P-value
Age (years)	− 0.170 ± 0.05	0.001	Age (years)	− 0.19 ± 0.05	0.024
Aortic annulus (mm)	− 0.799 ± 0.22	< 0.001	Aortic annulus (mm)	− 0.46 ± 0.28	0.097
Sinotubular junction (mm)	− 0.532 ± 0.15	< 0.001	Sinotubular junction (mm)	− 0.16 ± 0.29	0.560
Ascending aorta (mm)	− 0.572 ± 0.15	< 0.001	Ascending aorta (mm)	− 0.04 ± 0.31	0.898

*BMI* body mass index, *BSA* body surface area, *DBP* diastolic blood pressure, *SBP* systolic blood pressure

### Comparison of major aortic nomograms for echocardiography in different populations

Comparing the findings in this study with the results of studies in different populations, it can be seen in Table 5 that the demographic data of the male population of this study was like those in other studies. Of note, however, the males in this study population were generally younger and were shorter in height when compared to the male populations of similar studies in other populations. The demographic data of the women in this study differed to a greater degree when compared to female populations in other studies (Table 6). The women in this study were of greater weight (> 15 kg) than the average weights of women in other studies, they were also shorter and had a higher BSA, SBP, DBP and heart rate than women in other studies.

**Table 5:** Comparing male demographic data from studies describing normal reference ranges of proximal aorta dimensions

Author	Meel et al.	NORRE [1]	TAHES [12]	EMINCA [7]	Choi et al*. *[9]	Vriz et al*.* [6]
Age range	20–62	19–78	30–49	18–79	20–79	16–92
Age (years)	34.5 ± 10.2	48.0 ± 0.0	38.0	47.1 ± 16.2	48.0 ± 16.0	43.2 ± 16.2
Weight (kg)	72.3 ± 12.59	78.0 ± 0.0	58.5 (52–67)	67.6 ± 7.9	69.0 ± 9.0	77.9 ± 11.4
Height (cm)	166.0 ± 5.1	176.5 ± 0.0	167.0 (161–172)	171.0 ± 6.0	170.0 ± 7.0	175.8 ± 8.0
BSA (m^2^)	1.82 ± 0.2	1.9 ± 0.0	1.65 (1.52–1.78)	1.82 ± 0.1	1.8 ± 0.1	1.9 ± 1.2
SBP (mmHg)	126.5 ± 12.7	124.0 ± 0.0	119.0 (112–126)	121.0 ± 9.0	123.0 ± 12.0	127.8 ± 14.1
DBP (mmHg)	78.4 ± 12.7	77.0 ± 0.0	78.0 (73–84)	77.0 ± 7.0	75.0 ± 9.0	77.6 ± 9.4
HR (bpm)	66.2 ± 10.5	–	–	72.2 ± 8.5	68.0 ± 10.0	68.4 ± 12.7

*BSA* body surface area, *HR* heart rate, *DBP* diastolic blood pressure, *SBP* systolic blood pressure

**Table 6:** Comparing female demographic data from studies describing normal reference ranges of proximal aorta dimensions

Author	Meel et al.	NORRE [1]	TAHES [12]	EMINCA [7]	Choi et al*. *[9]	Vriz et al*.* [6]
Age range	20–62	19–78	30–46	18–79	20–79	16–92
Age (years)	38.8 ± 10.9	46.0 ± 0.0	35.0	47.5 ± 15.8	48.0 ± 16.0	46.0 ± 15.5
Weight (kg)	80.9 ± 18.7	63.0 ± 0.0	54.0 (48–63)	56.1 ± 6.6	56.0 ± 7.0	64.0 ± 9.1
Height (cm)	156.4 ± 6.1	163.0 ± 0.0	22.3 (19.5–25.3)	160.0 ± 5.0	158.0 ± 6.0	162.4 ± 6.7
BSA (m^2^)	1.86 ± 0.23	1.7 ± 0.0	1.54 (1.43–1.66)	1.6 ± 0.1	1.55 ± 0.1	1.7 ± 0.1
SBP (mmHg)	126.4 ± 11.6	117.0 ± 0.0	113.5 (107–121)	116.0 ± 11.0	118.0 ± 13.0	123.0 ± 14.7
DBP (mmHg)	79.9 ± 9.1	73.0 ± 0.0	77.5 (72–83.5)	74.0 ± 8.0	72.0 ± 10.0	75.1 ± 9.1
HR (bpm)	76.8 ± 11.7	–	–	72.6 ± 7.8	69.0 ± 9.0	72.9 ± 11.1

*BSA* body surface area, *HR* heart rate, *DBP* diastolic blood pressure, *SBP* systolic blood pressure

### Proximal aorta echocardiographic measurements

A comparison between the results of the aortic measurements found in this study to those in other studies can be found in Tables 7 and 8. Both the male and female populations in this study had generally smaller absolute values of aortic dimensions than those described in similar studies in different populations.

**Table 7:** Comparing male population data from studies describing absolute values of normal reference ranges of proximal aorta dimensions

Author	Meel et al.	NORRE [1]	TAHES [12]	EMINCA [7]	Choi et al*. *[9]	Vriz et al*.* [6]
Study design	Single centre (ASE guidelines)	Multi-centre, (I–I mid-systole)	Single centre (I–I mid-systole)	Multi-centre, (I–I end-diastole)	Multi-centre (L–L end-diastole)	Multi-centre (L–L end-diastole)
Location	South Africa	Europe	Benin	China	Korea	France, Italy, USA
Population	88(34 M)	704 (310 M)	513 (206 M)	1394 (678 M)	1003 (487 M)	1043 (503 M)
AA	19.7 ± 2.0	21.3 ± 2.0	21.4 (19.7–22.7)	21.3 ± 3.0	21.3 ± 1.8	21.0 ± 2.2
Aortic sinuses	28.1 ± 3.6	32.4 ± 3.7	28.5 (26.3–30.7)	30.1 ± 3.0	33.5 ± 3.2	31.8 ± 3.7
STJ	26.2 ± 3.38	27.2 ± 3.1	24.3 (22–26.6)	27.7 ± 4.0	27.3 ± 2.7	26.9 ± 3.7
AAO	25.7 ± 3.02	29.2 ± 3,6	27 (24.2–29)	24.4 ± 3.0	30.7 ± 3.7	29.1 ± 4.3

**Table 8:** Comparing female population data from studies describing normal reference ranges of proximal aorta dimensions

Author	Meel et al.	NORRE [1]	TAHES [12]	EMINCA [7]	Choi et al*. *[9]	Vriz et al*.* [6]
Study design	Single centre (ASE guidelines)	Multi-centre, (I–I mid-systole)	Single centre (I–I mid-systole)	Multi-centre, (I–I end-diastole)	Multi-centre (L–L end-diastole)	Multi-centre (L–L end-diastole)
Location	South Africa	Europe	Benin	China	Korea	France, Italy, USA
Population	88(54 F)	704 (394 F)	513 (307 F)	1394 (176 F)	1003 (516 F)	1043 (540 F)
AA	18.7 ± 2.5	19.2 ± 1.7	19.5 (18–20.6)	19.6 ± 2.3	19.4 ± 1.6	18.7 ± 1.6
Aortic sinuses	26.9 ± 7.3	28.9 ± 3.1	25.8 (24–27.5)	27.4 ± 3.1	30.1 ± 3.0	28.5 ± 3.0
STJ	24.7 ± 3.5	24.8 ± 2.7	22 (20–23.7)	25.9 ± 3.5	24.9 ± 2.7	24.4 ± 2.9
AAO	25.0 ± 3.6	26.9 ± 3.1	24.5 (22.8–26.7)	23.1 ± 3.5	29.1 ± 4.0	27.4 ± 3.4

*AA* aortic annulus, *AAO* ascending aorta, *STJ* sinotubular junction

## Discussion

This study reports normative values of echocardiographic measurements and circumferential strain of the proximal aorta in a healthy sub-Saharan African population.

Although the reference ranges for aortic dimensions are within the normal ranges outlined by the American Society of Echocardiography (ASE) Guidelines [3], they fall into the lower range of normality. This correlates with findings by LaBounty et al. whose study involving 15 295 adults provided evidence that people of Black African descent had smaller aortic diameters (both absolute and indexed for BSA) at the level of the aortic sinuses and ascending aorta than Caucasian, Asian, Hispanic and Native American counterparts [13].

Similarly, the absolute aortic diameters measured for males and females matched those recorded in the TAHES study (based on a West African population). Interestingly, despite the average BSA of the participants in this study, both male and female, being significantly greater than those in the TAHES study (1.65 m^2^ vs 1.82 m^2^ in men, and 1.54 m^2^ vs 1.86 m^2^ in females), the absolute aortic diameters in these two African based studies remain similar. The reason that people of African descent in general have smaller aortas could possibly be due to the relatively shorter height of African participants compared to Caucasian and Asian counterparts. However, due to data on normal values of proximal aorta diameters in people of African descent being scarce, with most existing data being derived from African American populations [12], more research is needed to shed further light on the cause behind this finding.

Absolute and indexed aortic diameters at the levels of the AA, sinuses, STJ and AAO all increased with increasing age in this study, with age being a significant independent predictor of diameter at the level of the ascending aorta. This correlates with findings in other major aortic dimension nomograms including the EACVI NORRE study [1]. Here it was found that there was an average increase of 0.33 mm/decade at the level of the AA, 1.31 mm/decade at the level of the sinuses (the greatest overall increase per decade), 0.62 mm/decade at the STJ and 0.82 mm/decade at the level of the AAO, these rates of increase per decade are like those reported by Mirea et al*.* [4]. The pathogenesis of aortic dilatation with increasing age relates to the progressive degeneration of the aortic wall due to decreased elastin content, elastin fractures, collagen deposition and calcification of the aortic media [14, 15]. This process is likely further exacerbated by subclinical pathological processes such as atherosclerosis [16, 17]. These processes ultimately result in an increase in aorta size, a loss of compliance and an increase in wall stiffness.

Further to this, in this study, it was found that age was the only significant independent predictor of aortic CS. The average change in circumferential strain per decade is 0.69% per decade, from a mean of 13.99 ± 5.27% in the age group of ≤ 29 years old to 9.62 ± 4.16% in the age group of ≥ 50 years old, [18] relating again to the degeneration of the vascular wall with age, wherein the stiffening and the widening of the aorta circumference results in a decrease in strain and thus a decrease in vascular compliance in the proximal aorta with age.

In the current study, males had consistently larger absolute and indexed aortic diameters than women, despite females in this study having a larger BSA than males. Male sex was also the strongest independent predictor of increased diameter at the level of the aortic annulus and sinotubular junction. These findings are most likely due to men having a significantly larger height than women (166.0 ± 5.1 cm in males vs 156.4 ± 6.10 cm in females).

On average, there was no significant difference in circumferential strain between men and women in the study which mirrors findings by Oishi et al*.* in 2011 [18]. However younger women (< 29 years) had higher aortic CS than younger males, while older women (> 40 years) had lower aortic CS than older males, which is consistent with findings by Waddell et al. [19]. As there is evidence that oestrogen affects connective tissue structure by slowing down the natural reduction of arterial compliance [20], hormonal changes later in life might be account for this finding.

Body surface area (BSA) was found to be a significant predictor of aortic diameter size at the level of the sinuses, sinotubular junction and at the level of the ascending aorta. BSA as a predictor of aortic root and ascending aorta dimensions is well documented [21]. The excess fat mass associated with obesity is known to increase metabolic demand and, thus, both cardiac output and total blood volume are elevated in obesity. These circulatory changes cause left ventricular geometric remodelling in the form of cavity dilatation, a structural change commonly seen in obesity, which is then thought to lead to a compensatory left ventricular hypertrophic response in response to increased wall stress [22].

In this study females also had a greater weight, body surface area (BSA), body mass index (BMI) and heart rate and were shorter than males. When compared to other similar studies, females in this study were of a greater weight (> 15 kg), were shorter in height and had a higher BSA, SBP, DBP and heart rates than women in other studies. However, despite BSA being a strong predictor of increased aortic diameters, and the BSA of women in this study being larger than other nomograms, aortic diameters were smaller than those in females in European and American studies. This further emphasises the effect of ethnicity playing a determining role in the smaller aortic dimensions seen in this study population. Regarding aortic CS, increasing BSA was associated with a decrease in aortic CS, however, this was not to a significant level.

This study provides supportive evidence that (i) ethnicity does influence echocardiographic measurements of the proximal aorta. Although these differences are relatively small, they could result in underdiagnosis or overdiagnosis of aortic dilatation in some individuals when using reference ranges derived from studies with unknown racial diversity (ii) That sex, BSA and age are significant predictors of aortic dilatation at different levels of the proximal aorta and (iii) that aortic CS decreases with age and with increasing ascending aortic diameter.

## Limitations

In terms of the limitations of this study, the study was designed to be homogenous in terms of race and ethnicity, therefore limiting the applicability of the normative values to alternative populations. Additionally, the small sample size and the higher average weight normal in this study population are additional considerations when interpreting and applying the data to other population groups.

In addition, patients with significant diseases such as diabetes and hypertension were excluded based on past medical histories obtained from the study participants and results of blood investigations or other clinical tests were not obtained, therefore patients with preclinical hypertension or subclinical disease might be included in the study, however, the effects of these states on the structures of the heart are not likely to be significant. Furthermore, interobserver variability may have affected the measurement of echocardiographic parameters, however, standard deviations of measurements were small and like those reported in other studies and so interobserver variability influence was negligible. The absolute values of aorta CS are subject to inter-vendor differences.

## Conclusion

This study provides normal reference ranges for dimensions of the proximal aorta and aortic circumferential strain in an African population. It serves as a platform for future larger studies and will allow the interpretation of aortic pathology in an African population. Further, circumferential strain serves as a marker for subclinical disease and can serve as a tool for early disease detection and cardiovascular risk factor modification.

## Data Availability

The datasets used and/or analysed during the current study are available from the corresponding author upon reasonable request.
